# Personalized 3D-Printed Prostheses for Bone Defect Reconstruction After Tumor Resection in the Foot and Ankle

**DOI:** 10.3390/jfb16020062

**Published:** 2025-02-11

**Authors:** Chang-Jin Yon, Byung-Chan Choi, Jung-Min Lee, Si-Wook Lee

**Affiliations:** Department of Orthopedic Surgery, Keimyung University Dongsan Hospital, Keimyung University School of Medicine, Daegu 42601, Republic of Korea; poweryon@dsmc.or.kr (C.-J.Y.); bcchoikr@gmail.com (B.-C.C.); biomechljm@gmail.com (J.-M.L.)

**Keywords:** three-dimensional printing, orthopedic oncology, patient-specific prosthesis, bone defect reconstruction

## Abstract

Three-dimensional (3D)-printing technology is revolutionizing orthopedic oncology by providing precise, customized solutions for complex bone defects following tumor resection. Traditional modular endoprostheses are prone to complications such as fretting corrosion and implant failure, underscoring the need for innovative approaches. This case series reports on three patients treated with 3D-printed, patient-specific prostheses and cutting guides. Preoperative CT and MRI data were used to design implants tailored to each patient’s anatomy, manufactured using electron beam melting technology with a titanium–aluminum–vanadium alloy. Functional outcomes showed significant improvements: in Case I, AOFAS improved from 71 to 96, and VAS decreased from 6 to 1; in Case II, AOFAS increased from 65 to 79, and VAS decreased from 5 to 3. Radiographic evaluations demonstrated stable prosthesis placement and early evidence of bone integration in Cases I and II, while in Case III, localized disease control was achieved before systemic progression. This case series highlights the transformative potential of 3D-printed prostheses in addressing the challenges of reconstructing anatomically complex defects. By enabling precise tumor resection and improving functional outcomes, this approach can advance current practices in orthopedic oncology. Further research should explore larger cohorts and use cost-effectiveness analyses to validate these findings and facilitate broader clinical adoption.

## 1. Introduction

Three-dimensional (3D) printing, first introduced in the 1980s, has revolutionized various industries, including medicine. Among medical applications, orthopedic oncology has emerged as a field that actively utilizes 3D printing for the management of both benign and malignant tumors [1,2,3,4]. A critical challenge in orthopedic oncology is addressing large bone defects following tumor resection, which are a major determinant of the success of limb salvage surgery [5,6]. Despite advancements in reconstructive techniques, current methods often fail to achieve both a precise anatomical fit and long-term mechanical stability. This unmet need underscores the importance of innovative approaches such as the use of 3D-printed, patient-specific prostheses. Traditional reconstructive options, such as allografts, autografts, combined grafts, segmental bone transport, bone cement spacers, and intercalary endoprostheses, each come with limitations in terms of precision, durability, and functional outcomes [7,8].

A modular endoprosthesis, often requiring intraoperative assembly, remains a common choice for reconstructing bone defects involving adjacent joints. However, these implants, particularly those with metal-on-metal (MoM) junctions, are prone to complications such as fretting and crevice corrosion. These phenomena can lead to implant failure, chronic inflammation, and adverse local tissue reactions, including pseudotumor formation and metallosis [9,10,11]. Recent studies, such as an investigation of cell-accelerated corrosion (CAC) of a CoCrMo alloy with segregation banding, have further highlighted the susceptibility of MoM implants to accelerated corrosion under physiological conditions, particularly in hip implant applications [12]. Unlike traditional modular endoprostheses, which require intraoperative adjustments and are prone to complications such as fretting corrosion and implant failure, 3D-printed patient-specific prostheses are pre-designed to fit a patient’s unique anatomy. This eliminates the need for extensive intraoperative modifications, reduces the risk of implant failure, and ensures precise anatomical reconstruction. The clinical significance of patient-specific 3D-printed prostheses lies in their ability to address unique challenges in orthopedic oncology, particularly for anatomically complex bone defects for which traditional implants often fail to achieve long-term stability and functional restoration. By offering a precise anatomical fit and reducing the need for intraoperative adjustments, this technology has the potential to revolutionize reconstructive surgery and improve quality of life for patients undergoing limb salvage procedures.

3D printing provides several advantages over traditional methods, including improved precision in bone cutting and implant fitting, reduced intraoperative time, and the ability to customize prostheses for anatomically complex defects. These benefits have been shown to minimize surgical complications and improve functional outcomes in limb salvage surgeries. Studies have demonstrated that 3D-printed PSI cutting guides significantly reduce bone-cutting errors, improve surgical precision, and enhance functional outcomes in limb salvage surgeries [13,14,15,16].

This report highlights a novel, patient-specific approach to reconstructing large bone defects after lower-extremity tumor resection. By integrating advanced imaging techniques, CAD software, and electron beam melting technology, we aimed to achieve precise anatomical reconstruction and improved functional outcomes in anatomically challenging cases. Seeking to address these challenges, we hypothesized that 3D-printed, patient-specific prostheses could enhance surgical precision, minimize complications, and improve functional outcomes in complex bone defect reconstructions. The objectives of this study were to (1) evaluate the feasibility of using 3D-printed prostheses in limb salvage surgery, (2) assess functional and radiographic outcomes, and (3) explore the potential advantages of this approach compared to traditional methods.

## 2. Materials and Methods

### 2.1. Patient Selection

This study included three patients with bone tumors around the foot and ankle who were chosen to undergo surgical resection and reconstruction using 3D-printed, patient-specific prostheses. The patient cohort comprised a 17-year-old female with a diffuse calcaneal neurofibroma (Case I), a 57-year-old female with low-grade chondrosarcoma of the distal tibia (Case II), and an 81-year-old male with a metastatic lesion of the distal fibula secondary to lung cancer (Case III). Preoperative assessments included detailed clinical evaluations, radiographic imaging (plain radiographs, CT, and MRI), and, where applicable, histopathological diagnosis.

### 2.2. Preoperative Planning and Design

The patient-specific prostheses and cutting guides were designed based on preoperative CT and MRI data, utilizing established methodologies to ensure anatomical precision and surgical accuracy [13,14]. Preoperative CT and MRI data were imported into segmentation software (Mimics 17.0, Materialise, Leuven, Belgium) to construct detailed 3D models of the bone and tumor regions. Segmented data were refined using computer-aided design (CAD) software (3-matics 9.0, Materialise, Leuven, Belgium) to create personalized prostheses and cutting guides tailored to each patient’s anatomy. Cutting guides were designed to enhance surgical precision, ensuring proper resection margins and alignment for prosthesis placement.

### 2.3. Prosthesis Fabrication

Prostheses were fabricated via electron beam melting (EBM) technology (ARCAM A1, ARCAM AB, Mölnlycke, Sweden) using titanium–aluminum–vanadium alloy (Ti6Al4V-ELI). The design process accounted for anatomical variations, mechanical load requirements, and integration with host bone. Prosthesis production, including post-processing to remove support material and ensure precision, was completed within 2–3 weeks. The implants and surgical guides were manufactured and certified by the MEDYSSEY Company (Jecheon, Korea) in compliance with regulatory standards [17].

### 2.4. Surgical Techniques

Each patient underwent tumor resection and reconstruction under general anesthesia. For Case I, involving a calcaneal neurofibroma, a medial heel incision allowed radical tumor excision, followed by the placement of a custom calcaneal prosthesis. In Case II, involving a distal tibial chondrosarcoma, a longitudinal medial ankle incision enabled intralesional curettage, cortical window creation, and cavity reconstruction with a pre-designed prosthesis and fixation plate. For Case III, involving a metastatic distal fibular lesion, a lateral fibular incision facilitated en bloc tumor excision, followed by reconstruction with a custom prosthesis ensuring clear resection margins.

### 2.5. Postoperative Evaluation

Postoperative evaluation included both functional and radiographic assessments. Functional outcomes were measured using standardized scoring systems: the American Orthopaedic Foot and Ankle Society (AOFAS) score, Visual Analog Scale (VAS), and the Musculoskeletal Tumor Society (MSTS) score. Radiographic parameters, such as prosthesis alignment, integration, and bone tissue growth, were assessed using plain radiographs and CT scans at regular follow-up intervals. Bone tissue growth and prosthesis stability were evaluated by observing radiographic evidence of cortical bridging, trabecular continuity, and absence of radiolucent zones around the prosthesis.

### 2.6. Ethical Approval and Consent

This study was conducted as a prospective research project approved by our institution’s institutional review board (IRB) under protocol number DSMC 2019-07-001-002. All patients provided informed consent prior to their participation in the study, including consent for the use of their medical data and imaging for research purposes. Our research adhered to the ethical principles outlined in the Declaration of Helsinki and institutional guidelines, ensuring patient safety and data confidentiality. The prostheses used in this study were investigational devices, custom-made for clinical research purposes and approved by the institutional review board (IRB). These devices were not produced under commercial regulatory frameworks like Medical Device Regulation (MDR) but followed strict institutional and ethical standards [18].

## 3. Results

### 3.1. Case I: Diffuse Neurofibroma of the Calcaneus

**Figure 1:**
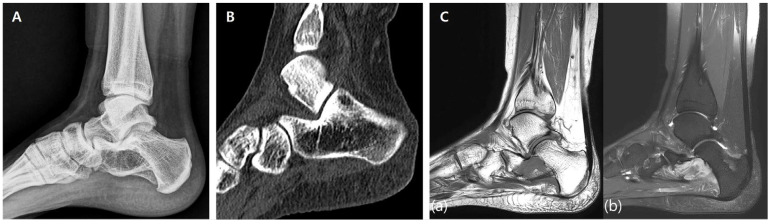
Preoperative imaging. (**A**) Preoperative X-ray showing a well-demarcated lesion within the calcaneus with cortical thinning and intact surrounding bone structures. (**B**) Preoperative CT scan demonstrating a 25 × 12 × 15 mm hypodense lesion in the calcaneal body, with thinning of the cortical bone and no evidence of periosteal reaction. (**C**) Preoperative MRI. (**a**) T1-weighted image showing homogenous low signal intensity. (**b**) T2-weighted image showing heterogeneous high signal intensity with central low-signal areas.

A 17-year-old female presented with a palpable mass on the medial aspect of her left foot and progressive difficulty in ambulation and daily activities. A family history of neurofibromatosis, specifically with respect to her mother, was noted. A preoperative radiographic evaluation revealed a 25 × 12 × 15 mm^3^ hypodense mass located in the body of the calcaneus. Plain X-ray (Figure 1A) and CT scans (Figure 1B) showed cortical thinning and a well-defined lesion within the calcaneus. MRI scans demonstrated a homogenous low signal intensity in T1-weighted images and heterogeneous high signal intensity with central low-signal areas in T2-weighted images, consistent with a benign neurogenic tumor (Figure 1C).

**Figure 2:**
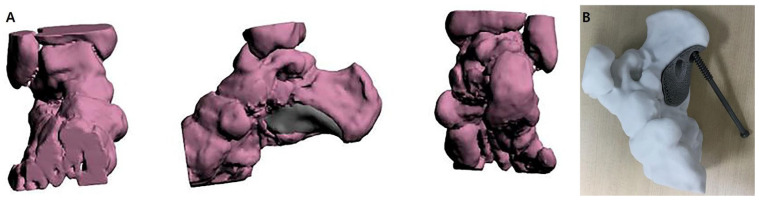
3D Surgical planning. (**A**) 3D image rendering from CT data used to simulate the calcaneal defect and design a personalized prosthesis for surgical reconstruction. (**B**) Clinical photograph of the 3D-printed prosthesis used for simulation.

Preoperative 3D image rendering was generated based on CT data to assess the calcaneal defect and aid in designing a patient-specific prosthesis for reconstruction (Figure 2A). The rendered model was used for the 3D printing of a personalized prosthesis (Figure 2B).

**Figure 3:**
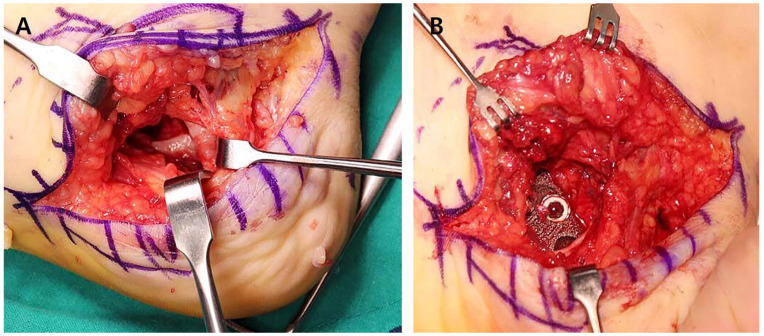
Intraoperative findings. (**A**) Clinical photograph showing the calcaneal defect after excising the tumor via an oblique incision on the medial heel. (**B**) Intraoperative view of the implantation of the pre-designed personalized prosthesis into the calcaneal defect.

**Figure 4:**
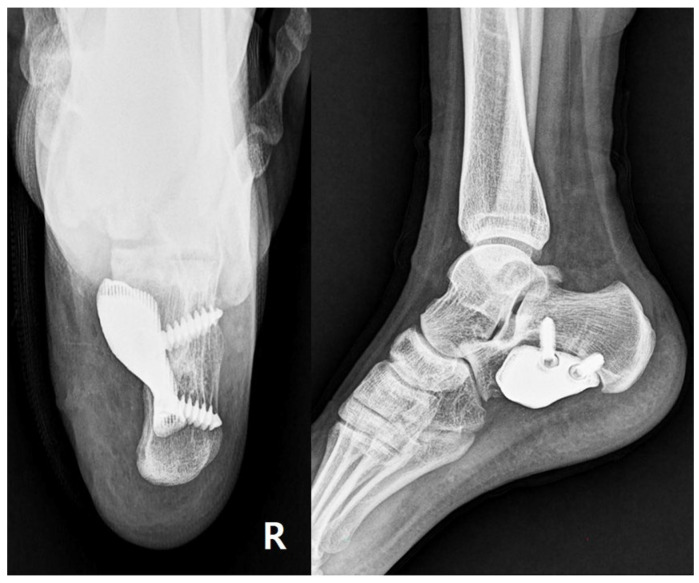
Postoperative imaging. Postoperative radiographs of the calcaneus of right foot in axial and lateral views showing stable fixation and prosthesis placement.

The patient underwent radical tumor resection via a 5 cm oblique incision on the medial heel. The surgical site revealed a significant calcaneal defect post-tumor excision (Figure 3A). The pre-designed prosthesis was implanted to reconstruct the defect (Figure 3B). Postoperative radiographs showed stable fixation with appropriate prosthesis placement (Figure 4).

Histopathological examination revealed spindle cell morphology, and the results of immunohistochemistry analysis were positive for S-100, confirming the diagnosis of diffuse neurofibroma. No neurologic deficits were noted postoperatively. At the two-year follow-up, the patient remained asymptomatic, with no recurrence observed. The AOFAS score improved from 71 to 96, the VAS score decreased from 6 to 1, and the MSTS score increased from 21 to 27. Radiographs at the 6-month follow-up showed stable prosthesis placement with no signs of loosening or radiolucency. Bone integration was evidenced by cortical bridging in the surrounding regions of the calcaneal prosthesis.

### 3.2. Case II: Low-Grade Chondrosarcoma of the Distal Tibia

**Figure 5:**
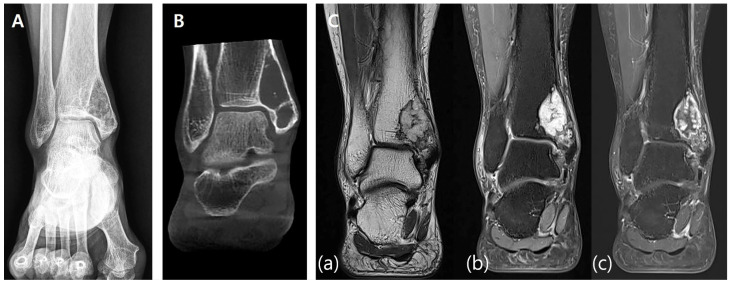
Preoperative imaging. (**A**) Preoperative X-ray demonstrating a lytic lesion with well-defined margins in the tibia. (**B**) Preoperative CT scan showing the lesion’s cortical involvement and surrounding bone structure integrity. (**C**) Preoperative MRI. (**a**) T1-weighted image displaying a hypointense lesion within the tibia. (**b**) T2-weighted image showing heterogeneous hyperintensity with internal low-signal regions. (**c**) Contrast-enhanced T1-weighted image highlighting the lesion’s vascular characteristics.

A 57-year-old female presented with right-ankle pain following a fall three weeks prior. Preoperative X-ray (Figure 5A) and CT scans (Figure 5B) demonstrated a heterogeneous hypodense mass measuring 15 × 27 × 41 mm, with irregular margins extending from the medial malleolus to the metaphyseal region of the distal tibia. MRI revealed a lobulated lesion with heterogeneous low-to-intermediate signal intensity in T1-weighted images, high signal intensity in T2-weighted images, and rim enhancement after contrast administration (Figure 5C). The lesion was provisionally diagnosed as low-grade chondrosarcoma based on the imaging characteristics observed.

**Figure 6:**
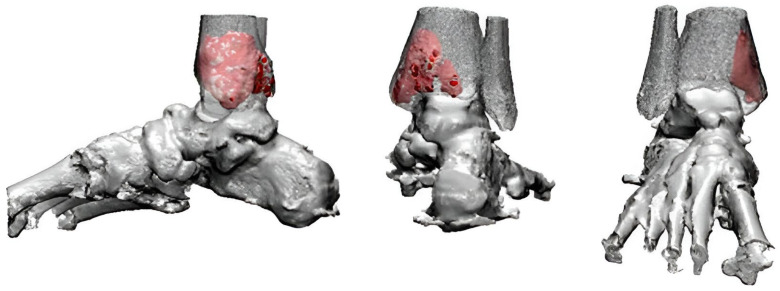
3D Surgical planning. Three-dimensional image rendering from CT data used for preoperative planning and simulation of prosthesis design.

**Figure 7:**
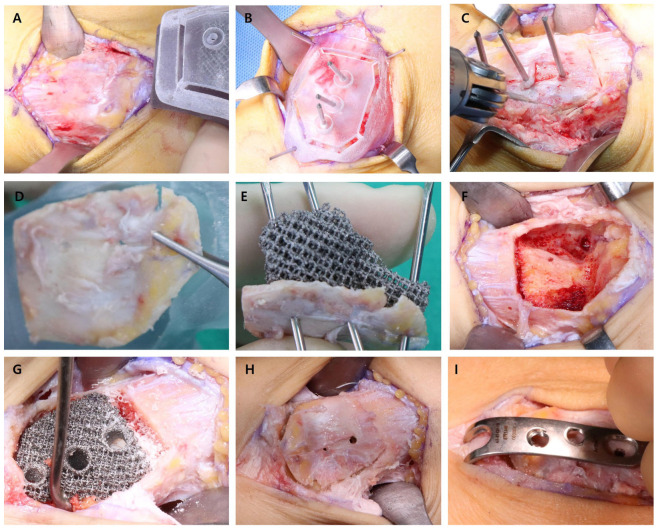
Intraoperative findings. (**A**) Intraoperative image showing the 3D-printed cutting guide being tested for proper size and fit after bone exposure. (**B**) Cutting guide secured to the tibia using K-wires for precise positioning. (**C**) Cortical bone resection performed using the cutting guide to create a well-defined cortical window. (**D**) Verification of the alignment of the cortical window with the cutting guide. (**E**) Patient-specific prosthesis tested for fit within the bone defect, with cortical bone temporarily fixed using K-wires. (**F**) Post-curettage view showing complete removal of the tumor within the tibia cavity. (**G**) Intraoperative insertion of the patient-specific prosthesis into the tibial bone defect. (**H**) Cortical window replaced over the prosthesis for anatomical reconstruction. (**I**) Final fixation using a medial malleolar plate to secure the cortical window and prosthesis.

Preoperative 3D surgical planning was conducted using CT data to create a personalized prosthesis and a cutting guide tailored for precise tumor resection and reconstruction (Figure 6). The patient underwent intralesional curettage through a 5 cm medial longitudinal incision. During the procedure, the 3D-printed cutting guide was first tested on the exposed tibia to confirm there was a proper fit (Figure 7A) and then secured with K-wires to ensure precise cortical window creation (Figure 7B). The cortical bone was resected along the guide, resulting in a well-defined cortical window (Figure 7C), which was subsequently verified for alignment with the guide (Figure 7D). Following complete tumor curettage, the patient-specific prosthesis was inserted into the bone defect (Figure 7F,G). Finally, the cortical window was replaced, and fixation was completed using a medial malleolar plate (Figure 7H,I).

**Figure 8:**
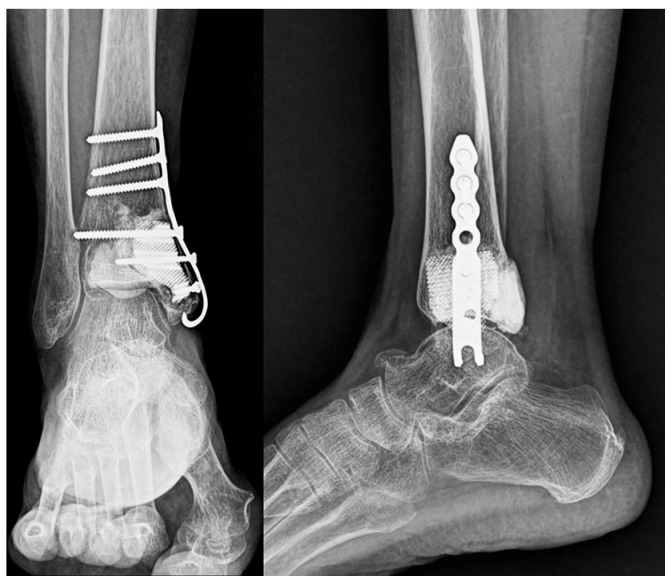
Postoperative imaging. Postoperative radiographs of the ankle in anteroposterior and lateral views showing stable fixation with a locking plate with proper prosthesis placement.

Postoperative radiographs confirmed stable prosthesis placement and proper alignment (Figure 8). The pathological findings were consistent with low-grade chondrosarcoma (Grade I).

Six months after the operation, the patient demonstrated significant improvements in functional outcomes, with the AOFAS score improving from 65 to 79, the VAS score decreasing from 5 to 3, and the MSTS score increasing from 20 to 22. Radiographic evaluations revealed stable prosthesis placement with partial integration and no signs of implant loosening or mechanical failure. However, follow-up CT scans revealed recurrence posterior to the initial lesion, necessitating re-curettage and cement insertion to address the defect. Pathology from the re-curettage again confirmed low-grade chondrosarcoma. At one year post-revision, the patient remained recurrence-free with stable prosthesis placement and no evidence of adverse events. Radiographs demonstrated further integration of the prosthesis into the surrounding bone, while the patient reported full functional recovery, including the ability to perform daily activities without restrictions.

### 3.3. Case III: Metastatic Fibular Lesion from Lung Cancer

**Figure 9:**
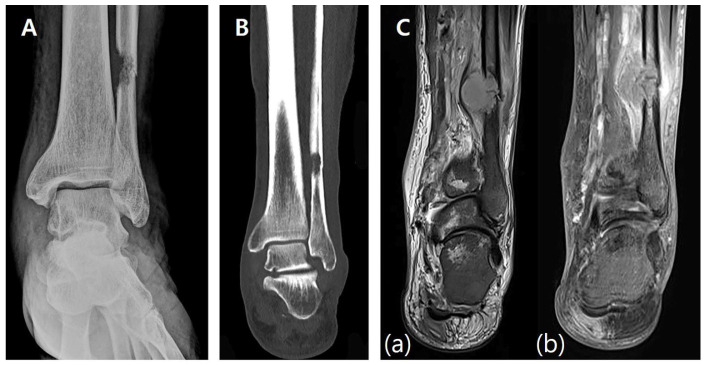
Preoperative imaging. (**A**) Preoperative X-ray showing a lytic lesion in the distal third of the fibula with cortical thinning and a visible fracture line. (**B**) Preoperative CT scan illustrating the lesion’s cortical destruction, dimensions, and evidence of a pathological fracture. (**C**) Preoperative MRI. (**a**) T2-weighted image demonstrating a hypointense lobulated mass with irregular margins. (**b**) Contrast-enhanced T1-weighted image showing heterogeneous intermediate-to-low signal intensity with central necrotic areas and prominent extraosseous extension.

An 81-year-old male presented with left-ankle pain following a fall. The patient had a history of stage-IIA non-small-cell lung carcinoma (NSCLC) treated with intensity-modulated radiation therapy nine months earlier. Preoperative imaging revealed a lytic lesion with cortical thinning and a fracture line in the distal third of the fibular shaft (Figure 9A,B). MRI revealed a 3.5 cm lobulated mass with central necrosis and prominent extraosseous extension. The signal characteristics were consistent with metastatic disease, as demonstrated via T2-weighted imaging showing a hypointense lobulated mass with irregular margins and through contrast-enhanced T1-weighted imaging displaying heterogeneous intermediate-to-low signal intensity with central necrotic areas and extraosseous extension (Figure 9C). Based on the imaging results and clinical history, the lesion was diagnosed as a pathological fracture secondary to metastatic lung cancer.

**Figure 10:**
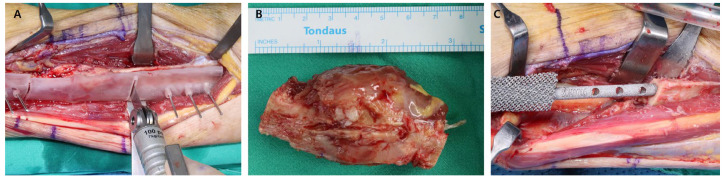
Intraoperative findings. (**A**) Personalized cutting guide fixed to the fibula, facilitating en bloc resection using a microsaw. (**B**) Clinical photograph of the tumor removed via en bloc resection. (**C**) Insertion of the 3D-printed patient-specific prosthesis into the bone defect following en bloc resection.

The patient underwent en bloc tumor excision via a 15 cm lateral fibular incision. A personalized cutting guide was fixed to the fibula to ensure precise resection, enabling en bloc removal using a microsaw (Figure 10A). The resected tumor measured approximately 3.5 cm and showed characteristic lobulated features with areas of necrosis (Figure 10B). Reconstruction of the fibular defect was achieved with a 3D-printed patient-specific prosthesis, which was precisely inserted into the defect following resection (Figure 10C). Pathological analysis confirmed metastatic squamous cell carcinoma with clear resection margins.

**Figure 11:**
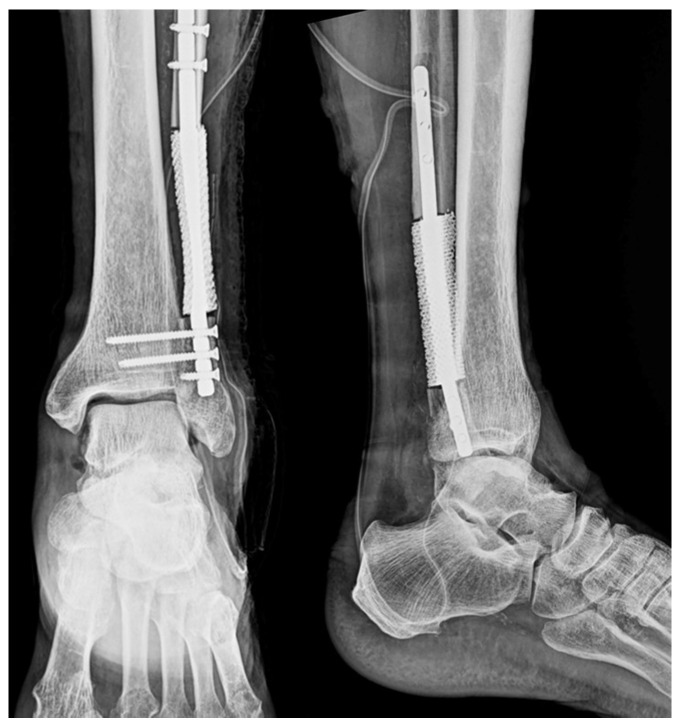
Postoperative imaging. Postoperative radiographs of the ankle in anteroposterior and lateral views showing stable intramedullary prosthesis fixation and proper alignment.

Postoperative radiographs showed accurate prosthesis placement and stable fixation with preservation of anatomical alignment (Figure 11). Although the surgery was successful, the patient experienced recurrence of the primary lung cancer three months after the operation and succumbed to obstructive pneumonia five months after surgery. Due to the patient’s metastatic disease, postoperative functional improvement was limited. However, radiographs confirmed accurate prosthesis alignment and stable fixation. Bone integration could not be assessed due to the patient’s limited survival period.

## 4. Discussion

This case series highlights the utility of 3D-printed patient-specific instruments (PSIs) in enhancing surgical precision and improving outcomes for complex tumor reconstructions. Functional outcomes (AOFAS, VAS, and MSTS) improved significantly for Cases I and II, reflecting the efficacy of PSIs in restoring mobility and reducing pain. For example, in Case I, AOFAS improved from 71 to 96, and VAS decreased from 6 to 1, while in Case II, AOFAS increased from 65 to 79, and VAS decreased from 5 to 3. Radiographic evaluations further confirmed stable prosthesis placement and early evidence of bone integration in both cases, highlighting the effectiveness of PSIs in achieving precise anatomical reconstruction. Radiographic evaluations demonstrated stable prosthesis placement and early evidence of bone integration in the majority of cases.

This study uniquely demonstrates the application of PSIs in anatomically complex regions like the foot and ankle, where a precise fit and mechanical stability are critical. This aligns with the findings acquired by Hayashi et al. [19], who emphasized the utility of PSIs in improving precision and stability in metastatic bone tumor surgeries. Although scaffold strength and detailed biomechanical properties were not directly measured in this study, the selection of titanium–aluminum–vanadium alloy provided sufficient structural support, based on the existing literature. Future studies should incorporate biomechanical testing and cost-effectiveness evaluations to validate the broader applicability of PSIs [20].

Neurofibromas, benign nerve sheath tumors, rarely undergo malignant transformation and are associated with a favorable prognosis. Habreal et al. successfully treated calcaneal intraosseous schwannoma with extended curettage and iliac bone grafting [21], while Ryan et al. reported patients were pain-free following the radical excision of distal tibial and fibular schwannomas without space filling [22]. In Case I, a personalized prosthesis was used to reconstruct a calcaneal defect after radical excision. This approach aligns with the findings of Habreal et al. [21], who demonstrated favorable outcomes using extended curettage and bone grafting in similar cases of schwannoma. The use of a personalized prosthesis in this study highlights a novel strategy for enhancing anatomical reconstruction and functional outcomes. By integrating PSIs into the surgical workflow, this case demonstrated the potential of personalized prostheses to restore function in anatomically challenging tumor resections.

The management of low-grade chondrosarcoma in long bones remains a subject of debate. Wide resection, while reducing recurrence, compromises function, whereas intralesional curettage is preferred for preserving limb functionality [23,24,25]. In Case II, the suspected low-grade chondrosarcoma was managed with intralesional curettage and reconstruction using a patient-specific prosthesis. Histopathological findings played a critical role in determining the extent of resection and the design of the prosthesis. For example, the low-grade chondrosarcoma required a conservative resection to preserve adjacent structures, which was reflected in the tailored design of the patient-specific prosthesis. This approach ensured adequate tumor clearance while minimizing functional impairment. Although local recurrence was noted, it remains unclear whether this resulted from incomplete initial resection or the inherent tumor biology of low-grade chondrosarcomas. Previous studies have reported recurrence rates of 5–20% in similar cases treated with intralesional curettage [25]. These findings underscore the importance of precise resection techniques and the potential ability of adjuvant therapies, such as cryotherapy or polymethylmethacrylate (PMMA), to extend resection margins and reduce recurrence risk [20,26,27,28]. Notably, the literature suggests that recurrence in low-grade chondrosarcoma does not significantly impact overall survival, aligning with the favorable outcome observed in this case [25]. While the application of personalized prostheses in this setting remains exploratory, the use of a pre-designed prosthesis provided an innovative solution for defect reconstruction, ensuring structural stability and preserving limb functionality. Future studies should explore how 3D-printed prostheses can be integrated with advanced adjuvant therapies to optimize oncological and functional outcomes. Further longitudinal research is also needed to evaluate the long-term impact of these approaches on recurrence rates and limb preservation.

For solitary bone metastasis, the aim of surgery is typically to alleviate pain, restore stability, and achieve oncological control [19]. In Case III, a metastatic fibular lesion secondary to non-small-cell lung carcinoma (NSCLC) was treated with en bloc tumor excision and reconstruction using a personalized prosthesis. Despite the patient’s poor systemic prognosis, the 3D-printed prosthesis enabled localized disease control, restored anatomical alignment, and alleviated pain. These short-term benefits likely contributed to an improved quality of life during the patient’s remaining lifespan, demonstrating the potential of 3D-printed solutions to provide meaningful functional and palliative benefits in patients with limited life expectancy. Postoperative radiographic evaluations confirmed the stability of the prosthesis and clear resection margins, aligning with the surgical objectives. Despite the success of the localized treatment, the patient succumbed to pneumonia within five months due to systemic progression of NSCLC. This case underscores the limited survival rates in metastatic disease, where the median survival time is often measured in months. However, it also highlights the potential of PSIs to provide significant short-term benefits, such as restoring anatomical integrity and alleviating pain, even in patients with limited life expectancy. By achieving precise resection and tailored reconstruction, personalized prostheses may represent a valuable option for improving the quality of life of patients with metastatic bone disease. Hayashi et al. similarly emphasized the importance of surgical precision and patient-specific approaches in improving outcomes for metastatic bone tumors, further supporting the findings of this study [19].

While this study showcases the innovative use of 3D-printed prostheses for addressing challenging bone defects following tumor resection, it is important to acknowledge its limitations. The small sample size and descriptive nature of this case series limit the generalizability of our findings. Additionally, the lack of long-term follow-up data restricts our ability to evaluate the durability and longevity of 3D-printed prostheses. These limitations highlight the need for further research with larger cohorts and extended follow-up periods. A key limitation is the lack of a cost-effectiveness analysis for 3D-printed prostheses [29]. While their clinical benefits are evident, their production costs and accessibility may pose barriers to widespread adoption. Furthermore, this study does not directly compare outcomes with those obtained using traditional methods, such as modular endoprostheses, which limits our ability to evaluate the relative advantages of 3D printing. Future research should address these aspects in controlled, comparative studies [30,31,32]. Specifically, larger cohorts and randomized controlled trials are needed to directly compare the outcomes for 3D-printed prostheses with those obtained using traditional methods, such as modular endoprostheses. Additionally, cost-effectiveness analyses should be conducted to assess the feasibility of the broader clinical adoption of 3D-printed solutions. These studies will provide critical insights into optimizing the use of patient-specific instruments in orthopedic oncology. Although this research was conducted as a prospective study, it is inherently experimental in nature due to the absence of a control group and its reliance on a case series design. This restricts the ability to draw definitive conclusions regarding the superiority of 3D-printed prostheses compared to traditional methods. Nonetheless, this study demonstrates the feasibility and potential of leveraging 3D printing to address the difficult problem of managing large bone defects after tumor resection. The production of 3D-printed prostheses involves advanced technology and expertise, potentially limiting their widespread availability. Ensuring consistent quality, meeting regulatory requirements, and addressing the learning curve associated with their surgical implementation are additional challenges that must be overcome to facilitate their broader clinical use. Additionally, the high production costs and limited availability of 3D-printing technologies serve as significant barriers to widespread adoption. Addressing these obstacles through cost-effectiveness analyses, streamlined manufacturing processes, and enhanced training programs will be essential to realize the full potential of 3D-printed prostheses in routine clinical practice. By providing anatomical accuracy and enhancing surgical precision, this approach represents a promising direction for future orthopedic oncology research.

While this study demonstrates the feasibility of using 3D-printed PSIs in complex reconstructions, it is inherently limited by its small sample size and a lack of biomechanical testing. These limitations restrict the generalizability of our findings and the ability to draw definitive conclusions about the superiority of 3D-printed PSIs over traditional methods. Veth et al. [18] have also noted that adjunctive treatments, such as cryotherapy and polymethylmethacrylate (PMMA), can enhance outcomes, suggesting that future research could explore combinations of these approaches with PSIs to optimize surgical success.

## 5. Conclusions

This case series underscores the transformative role of 3D printing in orthopedic oncology. Patient-specific instruments facilitated precise tumor resection and reconstruction, reducing surgical complexity and improving functional outcomes across diverse tumor types.

In orthopedic surgery, 3D-printed models are proving to be indispensable tools for joint replacement, tumor resections, and complex defect reconstructions. Addressing current financial and insurance barriers could enable broader adoption of these technologies. With their ability to personalize surgical solutions, patient-specific instruments represent a promising avenue for advancing the standard of care in orthopedic oncology.

## Data Availability

The datasets generated during the current study are not publicly available due to patient privacy laws but are available from the corresponding author on reasonable request.
